# Subclinical Epileptiform Process in Patients with Unipolar Depression and Its Indirect Psychophysiological Manifestations

**DOI:** 10.1371/journal.pone.0028041

**Published:** 2011-11-22

**Authors:** Petr Bob, Denisa Jasova, Jiri Raboch

**Affiliations:** Department of Psychiatry and UHSL, First Faculty of Medicine, Center for Neuropsychiatric Research of Traumatic Stress, Charles University, Prague, Czech Republic; Tokyo Metropolitan Institute of Medical Science, Japan

## Abstract

**Background:**

According to recent clinical findings epileptiform activity in temporolimbic structures may cause depressive and other psychiatric symptoms that may occur independently of any seizure in patient's history. In addition in these patients subclinical seizure-like activity with indirect clinical manifestations likely may occur in a form of various forms of cognitive, affective, memory, sensory, behavioral and somatic symptoms (the so-called complex partial seizure-like symptoms). A typical characteristic of epileptiform changes is increased neural synchrony related to spreading of epileptiform activity between hemispheres even in subclinical conditions i.e. without seizures. These findings suggest a hypothesis that measures reflecting a level of synchronization and information transfer between hemispheres could reflect spreading of epileptiform activity and might be related to complex partial seizure-like symptoms.

**Methods and Findings:**

Suitable data for such analysis may provide various physiological signals reflecting brain laterality, as for example bilateral electrodermal activity (EDA) that is closely related to limbic modulation influences. With this purpose we have performed measurement and analysis of bilateral EDA and compared the results with psychometric measures of complex partial seizure-like symptoms, depression and actually experienced stress in 44 patients with unipolar depression and 35 healthy controls. The results in unipolar depressive patients show that during rest conditions the patients with higher level of complex partial seizure like symptoms (CPSI) display increased level of EDA transinformation (PTI) calculated between left and right EDA records (Spearman correlation between CPSI and PTI is r = 0.43, p = 0.004).

**Conclusions:**

The result may present potentially useful clinical finding suggesting that increased EDA transinformation (PTI) could indirectly indicate increased neural synchrony as a possible indicator of epileptiform activity in unipolar depressive patients treated by serotoninergic antidepresants.

## Introduction

Recent clinical findings indicate that epileptiform activity in temporolimbic structures may cause depressive and other psychiatric symptoms that may occur independently of any seizure in patient's history [1]–[7]. Additionally there is evidence that many depressive and other psychiatric patients have positive clinical response to anticonvulsant treatment [2]–[5], [8], [9].

These findings are in agreement with the kindling hypothesis proposed by Post [10], who suggested that repeated depressive episodes in some patients may contribute to progressive changes that reduce the threshold needed to trigger recurrrent episodes. This increased vulnerability related to kindling may cause that the brain becomes more sensitized to the depressive state and the onset of future episodes is less related to stressful life events than at the beginning of the disease [11], [12]. This phenomenon, known as “kindling,” was first reported by Sevillano in 1961 and later elaborated by Goddard and other researchers, who comprehensively described it using electrodes stereotactically implanted into the amygdala and other brain regions [1], [13]. In this research, has been found that repetitive subthreshold stimuli elicit only small changes in EEG activity or behavior, whereas the same stimuli applied later in a state of increased sensitivity caused by the previous stimulation result in local after-discharges, increased synchronization, epileptiform EEG activity or seizures [1], [4], [13]. In this context, the concept of kindling as related to sensitization caused by stress or other stimuli may also be used as an explanation of the psychopathological processes that may mirror altered temporo-limbic functions due to epileptiform changes [1]–[4], [9], [10].

Together these findings suggest that in many cases depression and its pathogenesis may be closely related to an epileptiform process that may occur in temporolimbic structures frequently without any evidence on scalp EEG. Additionally there is evidence that this epileptic-like process may emerge in the form of symptoms similar to several symptoms of temporal lobe epilepsy (the so-called complex partial seizure-like symptoms) that occur as cognitive, affective, memory, sensory, behavioral and somatic symptoms [2], [4], [6], [13], [14]. These psychiatric presentations of epileptiform discharges likely are functionally linked to brain regions related to cognitive, affective and memory processes that are also involved in generation of temporal lobe seizures [2], [4]. These brain regions predominantly include limbic and paralimbic structures such as hippocampus, parahippocampal cortices and amygdala [15].

Although these symptoms typically occur in temporal epileptic patients and psychometric measures of the complex partial seizure-like symptoms were designed for epileptic patients, there is clinical evidence that clinically significant level of the same symptoms occur in non-epileptic patients as for example in patients with depression, schizophrenia, PTSD or in patients with traumatic brain injury [3], [5], [13], [14], [16]. In addition these non-epileptic patients likely display significant response to anticonvulsant drugs which strongly suggest that similar psychobiological mechanisms produce the seizure-like symptoms in epileptic as well as in non-epileptic patients included in the clinical group of the so-called Epilepsy Spectrum Disorders [2], [3], [5].

Although seizures in temporal lobe structures may occur only locally and unilaterally, frequently the seizure activity tend to increase neural synchronization and extend to the other hemisphere which significantly influences interhemispheric information transfer that may reflect temporal lobe “epileptogenicity” [17], [18]. These findings suggest a hypothesis that increased synchrony between hemispheres and interhemispheric information transfer may present physiological indicator that could distinguish depressive patients who frequently experience the complex partial seizure-like symptoms from those of them who experience these symptoms only rarely. Perspectively these findings could help to find clinical criteria that prospectively might be useful for indication of anticonvulsant treatment.

Because depressive patients predominantly have normal EEG and conventional scalp EEG is not able to provide information on subcortical structures, it is likely that direct measurement of limbic activity or its manifestations using EEG likely is not possible. Nevertheless there is evidence that epileptiform activity may manifest in the autonomic nervous system [19], [20] which suggests a possibility that subcortical discharges could be reflected through its influences on the autonomic nervous system.

With respect to this possibility recent evidence indicates that sensitive measure of autonomic changes reflecting brain functions presents bilateral electrodermal activity (EDA). The evidence shows that EDA is governed by ipsilateral limbic modulation influences and correlates with amygdala activity, although also other structures, such as the ventromedial and dorsolateral prefrontal cortices, anterior cingulate gyrus, parietal lobe, insula, and hippocampus are also involved in EDA modulation [21]–[23]. Evidence for the role of the amygdala in the expression of EDA mainly comes from functional-imaging studies and lesion studies [23], [24]. Further evidence provides intracranial data by Mangina and Beuzeron-Mangina [21], who reported that rapid and discontinuous changes in limbic EEG activity induced by electric stimulation are linked to rapid and discontinuous changes in EDA, which suggests a direct functional connection between limbic EEG activity and changes in EDA. In addition, there are also data suggesting that electrodermal activity as an indicator of the sympathetic arousal is able to reflect epileptic seizures [25].

Together these findings suggest a hypothesis that EDA could reflect epileptic-like conditions that are not directly presented on scalp EEG. In this context, it is possible to suppose that bilateral EDA could reflect increased synchrony and interhemispheric information transfer (transinformation) related to extension of epileptiform activity between left and right temporal lobe structures during resting conditions detected in EDA baseline activity that is not disturbed by outside stimuli.

## Methods

### Participants

For empirical examination of suggested hypothesis the methods of psychometric assessment (complex partial seizure-like symptoms, depression and momentarily experienced stress) and EDA measurement were used in 44 outpatients of the Charles university hospital (mean of age 37.06, age range 23–50, SD = 8.72) predominantly with high school education (mean of education 13.54 years). The patients had diagnosis of unipolar depressive disorder (26 patients with depressive episode and 18 patients with recurrent depression with mean period of depression 3.5 years), confirmed by clinical interview according to DSM IV criteria [26] and were also assessed by structured psychiatric interview M.I.N.I. version 5.0.0 [27]. The sample included 11 patients in remission, 25 in partial remission and 8 in relapse, with lasting depression less than 7 years and not more than 4 hospitalizations (average number of hospitalizations 1.5). Patients' treatment at the time of recruitment was based only on serotoninergic antidepressant medication in usual recommended doses.

With exception of age range (20–50), diagnosis (unipolar depression) and medication (serotoninergic antidepressants) and with exception of below mentioned exclusion criteria, there were not applied any selection criteria and the patients were assessed consecutively in order of their usual visits of the outpatient center.

Exclusion criteria for the patients were organic illnesses involving the central nervous system, sensory disorders, heart diseases, gastrointestinal disorders and other internal diseases, any form of epilepsy or epileptiform abnormalities on scalp EEG and mental retardation [IQ Raven higher than 90], psychotic disorders, electroconvulsive therapy, bipolar disorder, alcohol and drug abuse. Because high numbers of outpatients have unipolar depression and are treated by serotoninergic antidepressants we used this criterion for sample homogeneity. At this point the sample homogeneity was also criterion why we did not include unmedicated patients and patients with reactive depression, who may be momentarily influenced by stress factors.

With a purpose to compare the results from unipolar depresive patients we have included also 35 healthy controls (mean of age 35.12, age range 20–47, SD = 8.10). The control group involved 15 men and 20 women both predominantly with highschool education. The healthy controls were selected from general population that included hospital and university stuff members (N = 21) and university students (N = 14). All the controls were psychiatrically healthy according to M.I.N.I.

All the patients and controls gave written informed consent and the clinical study was approved by the university ethical committee.

### Psychometric measures

For the assessment of depressive symptoms Beck depression inventory- BDI-II [28] (in validated Czech version) was used. The BDI-II represents 21-items questionnaire for assessing depression where subjects indicate degree of their experience on 4-point Likert scale.

Complex partial seizure-like symptoms were assessed using complex partial seizure-like symptoms inventory– CPSI [2]. CPSI was originally designed to measure somatic, sensory, behavioral and memory symptoms associated with temporal lobe epilepsy (i.e. brief hallucinations, paroxysmal somatic disturbances, automatisms and dissociative disturbances). The inventory has 35 questions and subjects indicate degree of their experience on 6-point Likert scale (Cronbach's alpha 0.95, test-retest reliability after week 0.87). According to some data CPSI total score higher than 70 present a significant criterion for the so-called epilepsy spectrum disorder but also lower values may indicate an underlying electrophysiological dysfunction [2]. Although these symptoms were originaly described in patients with temporal lobe epilepsy, later studies have found that transient sensory, cognitive, and affective phenomena occuring in patients with complex partial seizures may be more common in patients with affective disorders and also in other psychiatric diseases than is usually known [3], [5], [6], [13], [14].

Symptoms of momentarily experienced stress were measured using Impact of Event Scale- IES [29]. IES is 15-items questionnaire and subjects indicate degree of their experience on 6-point Likert scale (Cronbach's alpha 0.85, test-retest reliability after week 0.81), reflecting intensity of posttraumatic phenomena, based on subjectively experienced stress. IES contains two main groups of questions which measure intrusion and avoidance.

### EDA measurement

EDA was recorded bilaterally using a two-channel SAM unit and Psylab software (Contact Precision Instruments) connected to a personal computer with sampling frequency 1000 Hz. The measurements were performed in a quiet room with a room temperature of about 23°C. During the measurement the participant was in resting state and sat in a comfortable chair. The measurement was performed using two pairs of Ag/AgCl electrodes (8 mm diameter active area) filled with electroconductive paste that were attached to the medial phalanges of the index and middle finger of each hand.

### Data analysis

Practical approaches to studying complex dynamical systems, such as the human brain, present methods of time-series analysis [30]. Data for this analysis may provide, for example, a psychophysiological measurement performed on the system during an experiment. Because observational data reflect only a few real independent variables of a system, approximation of the dynamic system behavior therefore uses a finite number of (mathematically reconstructed) variables to approximate states of the system or relationships between the subsystems. In this context it is possible to use a measure of the mutual interaction and information flow between subsystems that may be computed in the phase space coupling measures such as pointwise transinformation (PTI). This coupling measure takes into account also nonlinear dependencies and can be applied to nonstationary time series. The PTI of two observable quantities has been derived from Shannon's information concept and is calculated from the probability densities of the observables in the phase space [31]. The method may be applied to the recorded signals representing the complex couplings of the physiological subsystems (in this case left and right side of the EDA).

This method of nonlinear data analysis was applied to 100 seconds long left- and right- EDA time series during rest using algorithm for pointwise transinformation which is included in software package Dataplore. The algorithm is performed through calculation of transinformation between EDA time series x1 (left) and x2 (right). The pointwise transinformation is a time-resolving variant of the transinformation, returning an estimation of the transinformation for each sample (point in time).

The defining formula for the i-th time step is:
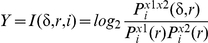
where the transinformation I is a function of the relative shift and relative phase space radius r, given for every sample point i. It denotes the probability to find a point of the reconstructed trajectory within a sphere of radius r around the i-th phase space point and refers to the phase spaces of the time series x1 and x2.

Statistical evaluation of PTI values (in bits), CPSI, BDI II and IES values was performed using software package Statistica version 8.0 and included descriptive statistics, Spearman correlations and nonparametric Mann-Whitney test for independent samples.

## Results

**Figure 1:**
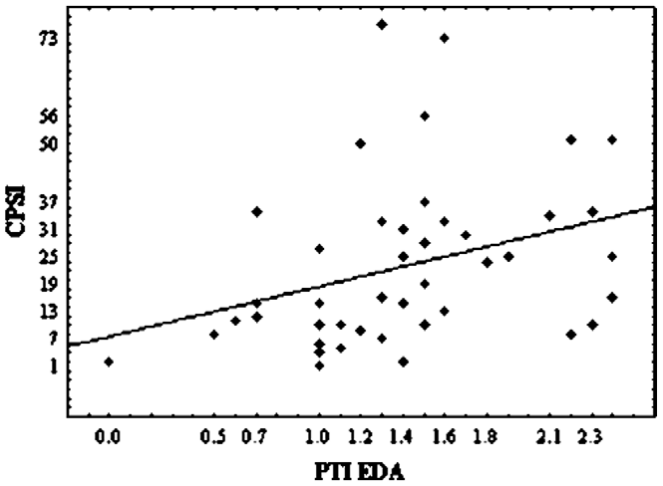
Dependency graph between pointwise transinformation- PTI (in bits) and complex partial seizure-like symptoms- CPSI (r = 0.43, p = 0.004).

The results show that during rest conditions complex partial seizure-like symptoms (CPSI) are significantly correlated with the information transfer (PTI) [r = 0.43, p = 0.004] (Figure 1). Other correlations between PTI and BDI-II (r = 0.07, p = 0.65), and between PTI and IES (r = 0.15, p = 0.34) were not statistically significant.

Additionally, we have used Mann–Whitney test for independent samples as a confirmation method for correlation analysis which indicate that the patients with higher level of complex partial seizure like symptoms (CPSI) display increased level of interhemispheric information transmission measured by PTI calculated between left and right EDA records in comparison to patients who have lower CPSI score (Table 1). Results in Table 1 also indicate that the levels of depression (BDI-II) as well as experienced stress (IES) are significantly higher in the group with increased CPSI.

**Table 1:** Between group comparison for depressive patients with higher and lower level of complex partial seizure-like symptoms.

	Mean low CPSI±S.D.	Mean high CPSI±S.D.	MW-testZ	p
**Age**	35.70±9.34	38.57±7.95	1.210	0.2262
**CPSI**	9.48±4.61	38.00±15.74	5.675	0.0000
**IES**	20.61±12.72	34.67±10.67	3.384	0.0007
**BDI-II**	13.83±7.71	24.95±14.38	2.819	0.0048
**PTI**	1.22±0.56	1.63±0.46	2.714	0.0066

***Note.*** CPSI- inventory for complex partial seizure-like symptoms; Higher CPSI (N = 9, CPSI≥16); Lower CPSI (N = 31, DES<16); IES- Impact of Events Scale; BDI-II- Beck Depression Inventory; PTI- pointwise transinformation (in bits); df = 42.

To distinguish the effect of BDI-II, IES and PTI on CPSI, we have used a multiple linear regression because it may be useful to know whether depression and PTI in their specific interactions are linked to increased levels of CPSI and whether BDI-II and PTI are tightly linked together. The result shows that multiple R = 0.76 is statistically significant (p<0.01; F = 17.93) which enables to define CPSI as a linear function of three variables CPSI = *f* (BDI-II, IES, PTI).

In further evaluation we have also performed correlational analysis of the psychometric measures with EDA activity (skin conductance level) for each patient on both hands. The results indicate that there is no direct association between EDA and psychometric measures and also we have not found any association between EDA changes and disease conditions such as the absence of differences between EDA in relapse and in remissions, as well as between pharmacotherapy response and EDA. In addition worthy of attention are also correlations between BDI-II and CPSI (r = 0.51, p<0.01) and between IES and CPSI (r = 0.55, p<0.01) that likely also may influence the PTI.

The same correlational analysis of the psychometric measures with PTI and EDA activity (skin conductance level) for each participant on both hands we have also performed in the healthy control group. The results indicate that there is no direct association between PTI, EDA and psychometric measures. Similarly as in depressive patients we have found statistically significant correlations between BDI-II and CPSI (r = 0.59, p<0.01) but not between IES and CPSI.

## Discussion

The results indicate that information flow between two EDA channels is related to complex partial seizure-like symptoms but not to depression or actual stress symptoms. With respect to current findings this result provides the first evidence on the relationship of EDA transinformation with complex partial seizure-like symptoms presented as cognitive, affective, memory, sensory, behavioral and somatic symptoms in depressive patients.

Findings of the present study are also in agreement with clinical data reported by several studies. For example, Silberman et al. [3] reported the occurrence of transient sensory, cognitive and affective changes that are very similar to symptoms described in patients with temporal lobe epilepsy in patients with affective illness and found that transient sensory, cognitive, and affective phenomena occuring in patients with complex partial seizures may be more common in patients with affective disorders and also in other psychiatric diseases than is usually known [3]. Varney et al. [5] reported similar study of depressive patients with well documented treatment resistance to tricyclic antidepressants, who frequently experienced multiple partial seizure like symptoms. They found that majority of these patients showed moderate or substantial improvement in their affective status in response to carbamazepin. In addition, they also found that reported complex partial seizure-like symptoms in these patients decreased significantly with treatment.

Similar result reflecting relationship between EDA transinformation (PTI) and psychosensory symptoms of temporal epilepsy we have found also in our previous study in alcohol dependent patients who during withdrawal and also in abstinent period in many cases display reduced inhibitory functions and kindling that likely may appear also in the form of psychosensory symptoms similar to temporal lobe epilepsy frequently in conditions of normal EEG and without seizures [32].

In this context, increased EDA transinformation could be explained by increased synchrony that may be caused by covered epileptic-like process, although this pathological process in principle does not mean that these patients have underlying neurological disorder, in fact epilepsy. With respect to close association of CPSI symtoms with stress (IES) and also with depression (BDI-II) and PTI it is likely that some symptoms of CPSI could reflect the increased emotional tension and stress at least in a subgroup of depressed patients. This assumption is in agreement with reported studies that repeated stressful stimuli may lead to increased vulnerability to stressors and influence sensitization with kindling-like progression that may result to limbic epileptiform activity [1], [4], [9], [10]. These epileptiform changes likely may be explained by some data that repeated stress leading to sensitization may cause changes in the subunit composition of the GABA-A supramolecular complex and several dopaminergic and serotoninergic changes in the amygdala that may lead to overstimulation of neurons in the amygdala and other limbic structures [4], [33]. These changes may be related to deficits in inhibitory functions and increased excitability of the limbic system manifesting as markedly increased prevalence of complex partial seizure-like symptoms.

Taken together these clinical findings are in agreement with evidence that focal seizures restricted to the hippocampus may progress without neurological clinical symptoms that are manifested when the focal hippocampal seizures spread to other structures such as the parahippocampal cortices and amygdala [15].

Partial limitation of this study is that we had not sufficiently large group of unmedicated unipolar depressive patients who could be included to the study. Inclusion of unmedicated patients in principle would be very useful for comparison of the results because SSRIs may cause various side effects that in principle could influence complex partial seizure-like symptoms and EDA transinformation. It is also possible that relationship between EDA transinformation and complex partial seizure-like symptoms could be stronger in unmedicated patients.

The relationship between EDA transinformation and complex partial seizure-like symptoms is also in agreement with findings suggesting that an important factor that implicates increase in neural synchrony and a possible transition of latent epileptiform process to clinical seizure is the speed of interhemispheric propagation of established epileptiform activity [17], [18]. This process is related to changes in interhemispheric information transfer that may not implicate clinical seizure because of interhemispheric inhibitory mechanisms that may reduce it to indirect clinical manifestations such as various forms of cognitive, affective, memory, sensory behavioral and somatic symptoms without full manifestations of epileptic clinical symptoms. Within this context relationship between complex partial seizure-like symptoms and EDA transinformation presents potentially useful clinical finding which could indirectly indicate spread of epileptiform activity between hemispheres that increases information transfer between them.

## References

[1] Kraus JE (2000). Sensitization phenomena in psychiatric illness: lessons from the kindling model.. J Neuropsychiatry Clin Neurosci.

[2] Roberts RJ, Gorman LL, Lee GP, Hines ME, Richardson ED (1992). The phenomenology of multiple partial seizure-like symptoms without stereotyped spells: an epilepsy spectrum disorder?. Epilepsy Res.

[3] Silberman EK, Post RM, Nurnberger J, Theodore W, Boulenger JP (1985). Transient sensory, cognitive and affective phenomena in affective illness. A comparison with complex partial epilepsy.. Br J Psychiatry.

[4] Teicher MH, Andersen SL, Polcari A, Anderson CM, Navalta CP (2003). The neurobiological consequences of early stress and childhood maltreatment.. Neurosci Biobehav Rev.

[5] Varney NR, Garvey MJ, Cook BL, Campbell DA, Roberts RJ (1993). Identification of treatment-resistant depressives who respond favorably to carbamazepine.. Ann Clin Psychiatry.

[6] Bob P (2003). Dissociation and neuroscience: history and new perspectives.. Int J Neurosci.

[7] Bob P (2008). Dissociation and neurobiological consequences of traumatic stress.. Activ Nerv Super.

[8] Johannessen LC (2008). Antiepileptic drugs in non-epilepsy disorders: relations between mechanisms of action and clinical efficacy.. CNS Drugs.

[9] Post RM, Weiss SR, Pert A (1988). Implications of behavioral sensitization and kindling for stress-induced behavioral change.. Adv Exp Med Biol.

[10] Post RM (1992). Transduction of psychosocial stress into the neurobiology of recurrent affective disorder.. Am J Psychiatry.

[11] Keller MB (2003). Past, present, and future directions for defining optimal treatment outcome in depression: remission and beyond.. JAMA.

[12] Monroe SM, Harkness KL (2005). Life stress, the “kindling” hypothesis, and the recurrence of depression: considerations from a life stress perspective.. Psychol Rev.

[13] Bob P, Palus M, Susta M, Glaslova K (2010). Sensitization, epileptic-like symptoms and local synchronization in patients with paranoid schizophrenia.. Prog Neuropsychopharmacol Biol Psychiatry.

[14] Bob P, Susta M, Gregusova A, Jasova D, Mishara A (2010). Traumatic stress, dissociation, and limbic irritability in patients with unipolar depression being treated with SSRIs.. Psychol Rep.

[15] McIntyre DC, Gilby KL (2008). Mapping seizure pathways in the temporal lobe.. Epilepsia.

[16] Roca V, Freeman TW (2002). Psychosensory symptoms in combat veterans with posttraumatic stress disorder.. J Neuropsychiatry Clin Neurosci.

[17] Weinand ME, Labiner DM, Ahern GL (2001). Temporal lobe seizure interhemispheric propagation time depends on non-epileptic cortical cerebral blood flow.. Epilepsy Res.

[18] Weinand ME, Hussain N, Labiner DM, Ahern GL (2006). Correlation of electrocorticographic to clinical seizure onset and interhemispheric propagation times in temporal lobe epilepsy.. Pathophysiology.

[19] Baumgartner C, Lurge RS, Leutmezer F (2001). Autonomic symptoms during epileptic seizures.. Epileptic Disord.

[20] Devinsky O (2004). Effects of seizures on autonomic and cardiovascular function.. Epilepsy Curr.

[21] Mangina CA, Beuzeron-Mangina JH (1996). Direct electrical stimulation of specific human brain structures and bilateral electrodermal activity.. Int J Psychophysiol.

[22] Phelps EA, O'Connor KJ, Gatenby JC, Gore JC, Grillon C (2001). Activation of the left amygdala to a cognitive representation of fear.. Nat Neurosci.

[23] Critchley HD (2002). Electrodermal Responses: What Happens in the Brain.. Neuroscientist.

[24] Furmark T, Fischer H, Wik G, Larsson M, Fredrikson M (1997). The amygdala and individual differences in human fear conditioning.. Neuroreport.

[25] Poh MZ, Loddenkemper T, Swenson NC, Goyal S, Madsen JR (2010). Continuous monitoring of electrodermal activity during epileptic seizures using a wearable sensor.. Conf Proc IEEE Eng Med Biol Soc.

[26] American Psychiatric Association (1994). American Psychiatric Association, DSM IV, Diagnostic and Statistical Manual of Mental Disorders..

[27] Sheehan DV, Lecrubier Y, Sheehan KH, Amorim P, Janavs J (1998). The Mini-International Neuropsychiatric Interview (M.I.N.I.): The development and validation of a structured diagnostic psychiatric interview for DSM-IV and ICD-10.. J Clin Psychiatry.

[28] Beck AT, Steer RA (1996). Beck Depression Inventory Manual..

[29] Horowitz M, Wilner M, Alvarez W (1979). Impact of Event Scale: A Measure of Subjective Stress.. Psychosom Med.

[30] Kantz H, Schreiber T (1997). Nonlinear time series analysis.

[31] Lambertz M, Vandenhouten R, Grebe R, Langhorst P (2000). Phase transitions in the common brainstem and related systems investigated by nonstationary time series analysis.. J Auton Nerv Syst.

[32] Bob P, Jasova D, Bizik G, Raboch J (2011). Epileptiform activity in alcohol dependent patients and possibilities of its indirect measurement.. PLoS One.

[33] Caldji C, Diorio J, Meaney MJ (2003). Variations in maternal care alter GABAA receptor subunit expression in brain regions associated with fear.. Neuropsychopharmacology.

